# A Biomimetic Basalt Fiber/Epoxy Helical Composite Spring with Hierarchical Triple-Helix Structures Inspired by the Collagen Fibers in Compact Bone

**DOI:** 10.3390/biomimetics7030135

**Published:** 2022-09-16

**Authors:** Jiahui Wang, Zhongyuan Shi, Qigang Han, Yanbiao Sun, Mingdi Shi, Rui Li, Rubin Wei, Bin Dong, Wen Zhai, Wenfang Zheng, Yueying Li, Nuo Chen

**Affiliations:** 1 Roll Forging Research Institute, School of Materials Science and Engineering (Key Laboratory of Automobile Materials, Ministry of Education), Jilin University, Changchun 130022, China; 2 Changchun Ruiguang Technology Co., Ltd., Changchun 130022, China; 3 Shandong Nonmetallic Materials Institute, Jinan 250031, China

**Keywords:** bone-inspired, hierarchical triple-helix structures, helical composite spring, lightweight, mechanical property

## Abstract

The lightweight property of helical composite spring (HCS) applied in the transportation field has attracted more and more attention recently. However, it is difficult to maintain stiffness and fatigue resistance at the same time. Herein, inspired by collagen fibers in bone, a bionic basalt fiber/epoxy resin helical composite spring is manufactured. The collagen fibers consist of nanoscale hydroxyapatite (increases stiffness) and collagen molecules composed of helical amino acid chains (can increase fatigue resistance). Such a helical structure of intercalated crystals ensures that bone has good resistance to fracture. Specifically, we first investigated the effect of adding different contents of NS to basalt fibers on the stiffness and fatigue properties of an HCS. The results show that the optimal NS content of 0.4 wt% resulted in 52.1% and 43.5% higher stiffness and fatigue properties of an HCS than those without NS, respectively. Then, two braided fiber bundles (TS-BFB) and four braided fiber bundles (FS-BFB) were designed based on the helical structure of amino acid chains, and the compression tests revealed that the maximum load resistance of TS-BFB and FS-BFB was increased by 29.2% and 44%, respectively, compared with the conventional single fiber bundle (U-BFB). The superior mechanical performance of TS-BFB and FS-BFB is attributed to the more adequate bonding of 0.4 wt% NS to the epoxy resin and the multi-fiber bundles that increase the transverse fiber content of the spring. The findings in this work introduce the bionic collagen fiber structure into the design for an HCS and provide a new idea to improve the spring performance.

## 1. Introduction

### 1.1. Bionic Spiral Structure and Composite Spring

In the evolution process of hundreds of millions of years, organisms in nature have gradually formed various materials with unique structures and functions to adapt to the natural environment. These materials, with their unique mechanical properties, can well meet the needs of organisms for various functions [1,2,3,4,5,6,7]. Among them, natural materials with helical structures are amazing and unique. For example, the outer part of the horns of male bovines is composed of α-keratin with a helical peptide chain, which is lightweight and has excellent energy absorption capacity [8]. The dactyl club of the mantis shrimp has a helical mineralized α-chitin fiber, which is an important factor for the dactyl club to withstand thousands of shocks [9]. The spiral structure can improve impact resistance and enhance stress dissipation [10]. The structure of the exoskeleton of the beetle elytron and the collagen fibrils of the scales of Arapaima gigas also present spirals [11,12]. A lot of research proved that the helical structure can provide high stiffness, strength, ductility, toughness, and damage resistance. Therefore, the bio-inspired helical material has great potential and research value and can be applied to the fields of automobiles, aviation, and the military [13].

A helical spring is a crucial component in automobile shock absorption devices. It can reduce the vibration and impact caused by an uneven road. A helical spring has many advantages, such as low natural vibration frequency, good damping effect, and the ability to improve the vehicle riding fatigue performance. With the characteristic of variable stiffness, the height of the car body can be automatically adjusted when it is used with a height-adjusting device. It has a long service life and is widely used in the shock absorption system of passenger vehicles which needs high comfort and stability [14,15,16,17]. With the promotion of hybrid and electric vehicles, automotive companies are particularly focused on improving properties by optimizing component structures. Traditional metal springs are heavy, have poor corrosion resistance, include impurities, and will break without warning when the force exceeds its failure load. Compared with metal, fiber-reinforced polymer composite materials have higher specific stiffness, excellent creep behavior, lighter weight, and excellent corrosion resistance. In addition, the crack propagation rate is very low, so it will not suddenly break like metal materials, which can improve the safety of vehicles when driving. At present, it has been applied in many parts of the automobile, such as the driving shaft, bumper, plate spring, etc. [18,19,20,21,22,23]. Therefore, it is of great significance to investigate the possibility of using fiber-reinforced composites to replace traditional metal materials.

For the application of fiber-reinforced polymer composites instead of steel, it is important to consider mechanical properties. To improve the mechanical properties of the Helical Composite Spring (HCS), many researchers have been working on the design and fabrication of composite suspension springs for automobiles. For instance, J.R. Pothnis et al. [24] reported a significant increase in the tensile properties of an epoxy polymer with the addition of 0.25 wt% carbon nanospheres. Aidin Mirzapour et al. [25] investigated whether the addition of nano-silica significantly improved the mass ablation rates and flexural strength of nanocomposites. Faruk Firat Calim [26] studied the dynamic behavior of the composite barrel and hyperboloidal springs. The results showed that a directly proportional relationship between the helix pitch angle and the vibration period is observed, but the effect of the helix pitch angle on the displacement amplitude is negligible. Oussama Zebdi [26,27] used a multi-objective optimization algorithm to calculate the optimal parameters for minimizing mass and maximizing the stiffness of an HCS. Ekanthappa J [28] verified that adding micron-size graphite powder into the continuous glass fiber reinforced polymer could increase the failure load of the HCS. Bok-Lok Choi [29] designed an HCS using continuous carbon fiber, which had excellent performance and lighter weight. However, the effect of nano-silica (NS) on the stiffness and fatigue performance of an HCS has not been studied, and few studies have applied the bionic ideas to the structural design of an HCS. Therefore, this study used NS modification treatment and prepared basalt fiber/epoxy HCS through extrusion molding, and discussed the influence of NS on the stiffness and fatigue performance of an HCS and the optimal NS addition amount. On this basis, HCS with the best performance after NS modification was selected and the idea of bionics was introduced to develop a new procollagen HCS. and Then, the effect of bionic structure on an HCS is discussed.

### 1.2. Tropocollagen-Inspired Basalt Fiber Reinforced HCS Model

**Figure 1:**
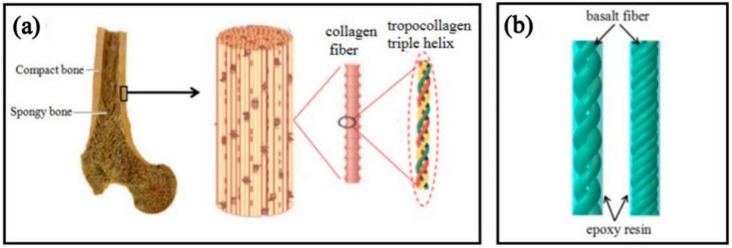
(**a**) Hierarchical structure of the bone [30]; (**b**) tropocollagen-inspired basalt fiber reinforced helical composite spring model.

Due to their excellent toughness, rigidity, strength, and special spiral structure, the tropocollagen fibers of compact bone inspire the design of new structural materials. Figure 1a shows the hierarchical structure of bone. The bones are comprised of compact bone on the surface, spongy bone, and its tissues inside. Compact bone is composed of collagen fibers and surrounding minerals, and the structural unit of collagen fibers is triple helix-shaped tropocollagen fibers. The three chain-like amino acids of procollagen fibers are arranged alternately, which balances the distribution of tropocollagen fibers in all directions in the bone and enhances the shear resistance of the bone [30,31,32]. In this study, we used continuous eco-friendly basalt fibers (basalt fibers have high strength, modulus, and chemical stability [33]) to fabricate a helical composite spring woven from fiber bundles (Figure 1b) by imitating the unique triple helix structure of tropocollagen fibers and tested its compression properties. The interwoven fiber bundles are like the amino acid chains arranged alternately by the tropocollagen fibers, and the epoxy resin wrapped on the surface of the fiber bundles is equivalent to the minerals distributed around the tropocollagen fibers.

## 2. Materials and Methods

### 2.1. Materials

Basalt fibers with a linear density of 2400tex, purchased from Tianlong Basalt Continuous Fiber Co., Ltd., Yangzhou, China, were used as reinforcement in the HCS. Epoxy resin YT-CC301 with hardener YT-CC302S (supplied from Yituo Composite Co., Ltd., Suzhou, China) was used as the matrix. NS with an average diameter of 30nm was purchased from Tonglin Chemical Co., Ltd., Zibo, China. It is an amorphous white powder that is spherical in microscopic view, non-toxic, odorless, non-polluting, and can improve the mechanical properties and anti-aging properties of mixed materials. Silane coupling agent KH550, supplied from Kangjin New Material Technology Co., Ltd., Dongguan, China, was used as a dispersant and dissolved in absolute ethanol for surface treatment of nano-silica fillers to improve the bonding and dispersibility in the resin matrix and improve the basalt fiber bonding performance with the resin matrix.

### 2.2. Fabrication of an HCS

**Figure 2:**
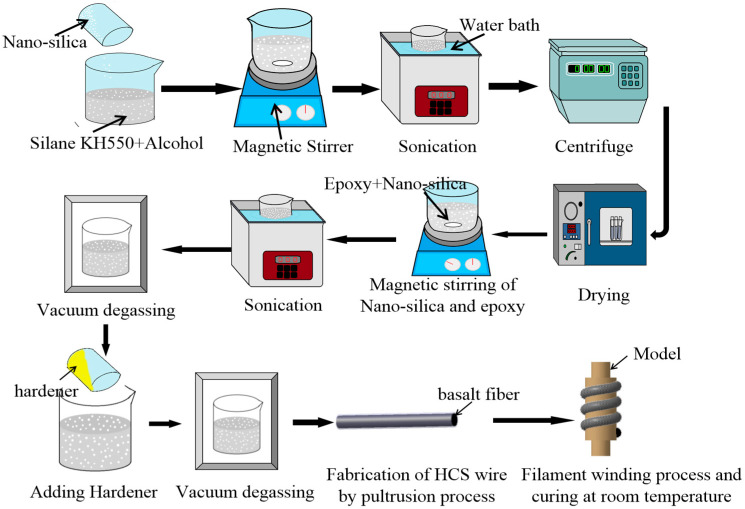
Process chart for preparing basalt fiber/epoxy helical composite spring.

The whole process is shown schematically in Figure 2. Nano-silica tends to agglomerate because of its high surface energy. To break the NS agglomerates, the nanoparticles were suspended in the mixture solution of silane coupling agent KH550 and ethanol and subjected to magnetic stirring for 40 min at first. Then, the suspension was subjected to bath sonication at room temperature for 30 min. Thirdly, the mixture solution was dialyzed by a high-speed centrifuge for 1 min. Next, the surface-treated NS was obtained by storing and grinding. The surface-treated NS was mixed with the epoxy and subjected to magnetic stirring at 40 °C for 40 min. After magnetic stirring, the suspension was sonicated using a sonicating bath for 30 min to ensure uniform dispersion of NS and subjected to vacuum degassing. The hardener was added to the suspension in the ratio of 3:10 by weight, and the air bubbles were removed from the solution. Then, the continuous basalt fibers were wetted by the application of a matrix and pulled into the hose by using pultrusion. In this step, basalt roving was directly pultruded into a fixed-size silicone hose to obtain a unidirectional fiber bundle(U-FB). If the spiral structure of collagen fiber was imitated, the same amount of basalt twists less roving was divided into two- and four-fiber bundles to obtain two strands of the braided-fiber bundle (TS-BFB) and four strands of the braided-fiber bundle (FS-BFB). Finally, the preformed HCS samples were winded onto a mold to be completely cured for 8 h at room temperature.

### 2.3. Performance Tests

**Figure 3:**
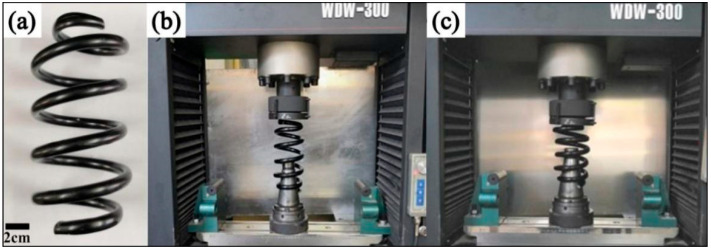
The HCS samples and test mechanism: (**a**) HCS samples; (**b**) test setup; (**c**) sample under testing.

Figure 3a shows the experimental samples of an HCS (height = 200 mm, diameter = 12 mm, medium diameter = 90 mm). The stiffness tests were conducted with an electronic universal testing machine (WDW-300) at a compression speed of 1 mm/min. Figure 3b,c shows the experimental setup for measuring the mechanical properties of the HCS samples. According to JIS B2704, the spring stiffness is determined by the two endpoints between 30–70% of the deformation under the test load.

The fatigue test of the spring was carried out in a TPJ-20 fatigue tester made by Jinan Kang Yuan Instrument Company in China, which was equipped with a counter for recording the number of experiments. The compression stroke was 50% of the free height, and the number of cycles was 1000. The effect of different content of NS on the structure of an HCS was investigated with the help of scanning electron microscopy. In addition, the samples were cut off 2–3 mm from the fracture surface to study the fracture mechanism. All scanned specimens were observed with a JSM-7900 scanning electron microscope produced by Japan Electronics Corporation, Tokyo, Japan after gold spraying treatment, and energy spectrum tests were also performed. The scanning voltage was set at 10 kV.

## 3. Results and Discussion

### 3.1. Performance Test of an HCS Modified by NS

#### 3.1.1. Stiffness Analysis

**Figure 4:**
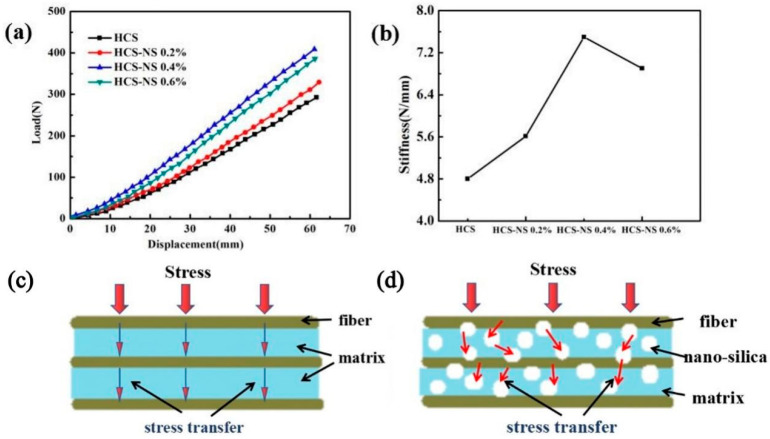
(**a**) Load-displacement curves of helical spring compression test; (**b**) the curve of static stiffness of helical spring; (**c**) schematic diagram of composite stress transfer of pure fiber composite; (**d**) schematic diagram of nano-silica-fiber-reinforced epoxy resin composite stress transfer.

Figure 4a is the load-displacement curve of the static stiffness tests of the HCS with different NS content, and Figure 4b is the calculated stiffness change curve of the HCS. It can be seen from the figure that as the NS content increased, the stiffness gradually increased. When the NS content was 0.4 wt%, the stiffness of the HCS reached the maximum. As shown in Table 1, the spring stiffness value increased from 4.8 N/mm to 7.3 N/mm, an increase of 52.1%. Rasol HA et al. [34] concluded that the stiffness of the carbon fiber spring is 5.66, and the HCS containing 0.4 wt% NS has a significantly higher stiffness than the carbon fiber spring. The excellent stiffness of the HCS with 0.4 wt% NS could be attributed to the combined results of the two mechanisms. Firstly, adding NS can increase the strength of the matrix. Secondly, proper wettability makes the interface between the matrix and the fiber have good adhesion. The superior matrix strength may be due to the effective transfer of stress from the matrix phase to the NS through the NS/epoxy interface. The fact that NS enhanced the strength of the epoxy resin matrix may be based on two reasons [35]: the bonding strength and the interface area of the NS/epoxy resin. The interface bonding strength of the NS/epoxy resin mainly depends on the various chemical, physical, and mechanical interactions between NS particles and epoxy resin. However, the NS/epoxy interface area is the main reason for adding NS content in fiber composites to increase the strength. The addition of NS promoted the formation of a high specific surface area so that the inside of the HCS wire had a very high interface area. The existence of such a large interface area enabled the stress to be transferred from the epoxy matrix to the NS particles (the schematic diagram of the stress transfer is shown in Figure 4c,d), which enabled the NS-reinforced fiber composites to withstand more force than unreinforced ones. In addition to the existence of interfacial stress transfer, due to the physical adsorption phenomenon [36], the adhesion of nanofillers on epoxy made them stronger than pure epoxy. 

When the NS content increased from 0.4 wt% to 0.6 wt%, the stiffness of the HCS decreased by 10.6%. This could be attributed to the fact that nanofillers have a very high specific surface area leading to high surface energy. The high content of nanofillers in the epoxy matrix tended to form agglomerates. The formation of agglomerates would inevitably reduce the effective aspect ratio, resulting in a decrease in total free energy and a reduction in the availability of the net interface area. Therefore, the stress transfer became less effective, resulting in a decrease in the stiffness of the HCS. This is consistent with the conclusion that a gradual increase in NS content reduces the strength of the composite [37].

#### 3.1.2. Fatigue Analysis

**Figure 5:**
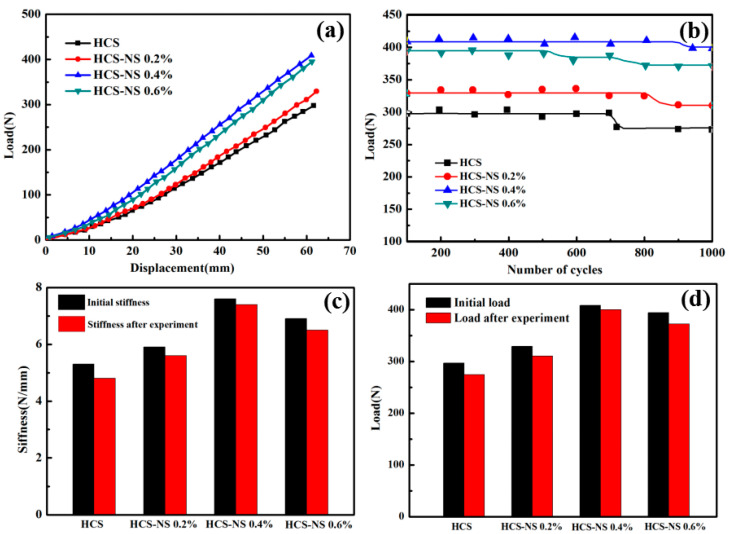
(**a**) The load-displacement curve during the static stiffness test; (**b**) the flat curves for the cycling test; (**c**) spring stiffness; and (**d**) compression load.

Figure 5a shows the cyclic loading process curve with an increment of 50% of the free height of the helical spring. It can be seen from the figure that the fiber and the matrix were repeatedly squeezed during the cyclic loading process, which would lead to the debonding of the fiber and the matrix and the generation of microscopic cracks in the internal defects of the matrix, resulting in a decrease in the bearing capacity. When the internal stress was released, the curve became flat again; As shown in Figure 5b, when the NS content is 0.4 wt%, the number of stable cyclic loading of the HCS is the largest, which is 43.5% higher than that of no NS. This could be attributed to the promotion of the addition of NS. The stress transferred from the matrix to the NS reduced the bearing pressure of the matrix and increased the toughness of the matrix. However, when the NS content increased from 0.4 wt% to 0.6 wt%, the stable cycle loading times decreased by 27.6%, and a secondary decrease occurred. The NS content in the HCS was too much, causing local nano-particle agglomeration. Due to the long-term reciprocating force and extrusion at the agglomeration site, which caused the stress concentration at the local particle agglomeration and generation of microcracks, the number of stable cyclic loading decreased. The reason for secondary decrease may be caused by microcracks in the matrix or other local aggregates. Figure 5c shows that the composite spring exhibits good stiffness after the addition of 0.4 wt% NS, and the stiffness of the spring remains high after cyclic loading. Figure 5d shows the change of the maximum load before and after cyclic loading. The load resistance of the four kinds of HCS with different NS content all decreased after cyclic loading, but the load dropped with the NS content of 0.4 wt% was the smallest. When the NS content exceeded 0.4 wt%, due to the local agglomeration of nanoparticles, the load-bearing capacity was significantly reduced compared to the one without NS. The degradation results are shown in Table 2. Although the anti-load capacity of the helical spring decreased after cyclic loading, there was no macro crack on the surface of the HCS. The decrease in the load-bearing capacity was mainly due to the release of the internal stress of the spring wire.

#### 3.1.3. Micro-Interface Analysis

**Figure 6:**
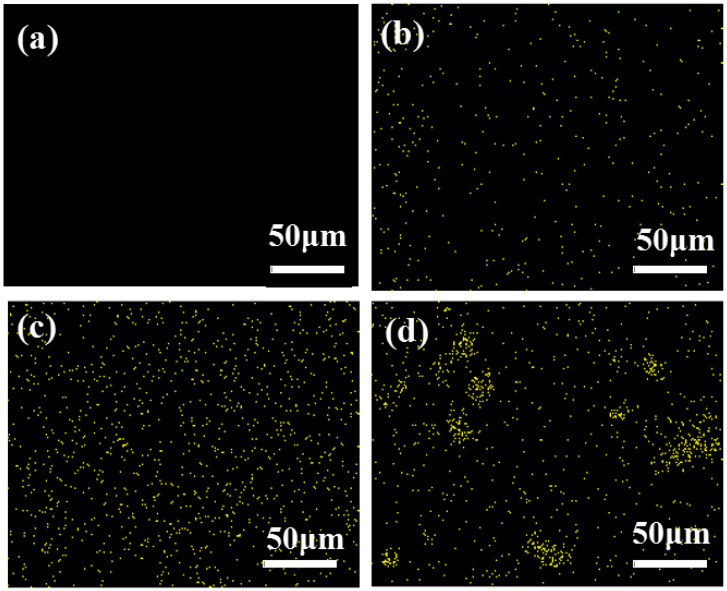
EDS images of Si of basalt fiber reinforced epoxy resin with different content of nano-silica: (**a**) 0 wt%; (**b**) 0.2 wt%; (**c**) 0.4 wt%; (**d**) 0.6 wt%.

Figure 6 shows the EDS mapping for Si in the basalt-fiber-reinforced epoxy resin. Compared to HCS without NS (Figure 6a) and NS content of 0.2 wt% (Figure 6b), it is clear from Figure 6c that at 0.4 wt% NS content in the composite, the nanoparticles throughout the matrix exhibit excellent dispersion, resulting in a large NS/epoxy interface area that tightly surrounds the basalt fibers to form a transition layer thereby increasing the density and modulus of the matrix [38]. This is an important factor leading to the increase of compression performance of the 0.4 wt% NS HCS compared to the HCS without NS. It is observed that the content of NS increased from 0.4 wt% to 0.6 wt%, and the NS lean towards forming agglomerates in local areas of composite (Figure 6d), resulting in a decrease in the overall free energy and the availability of net interfacial area.

**Figure 7:**
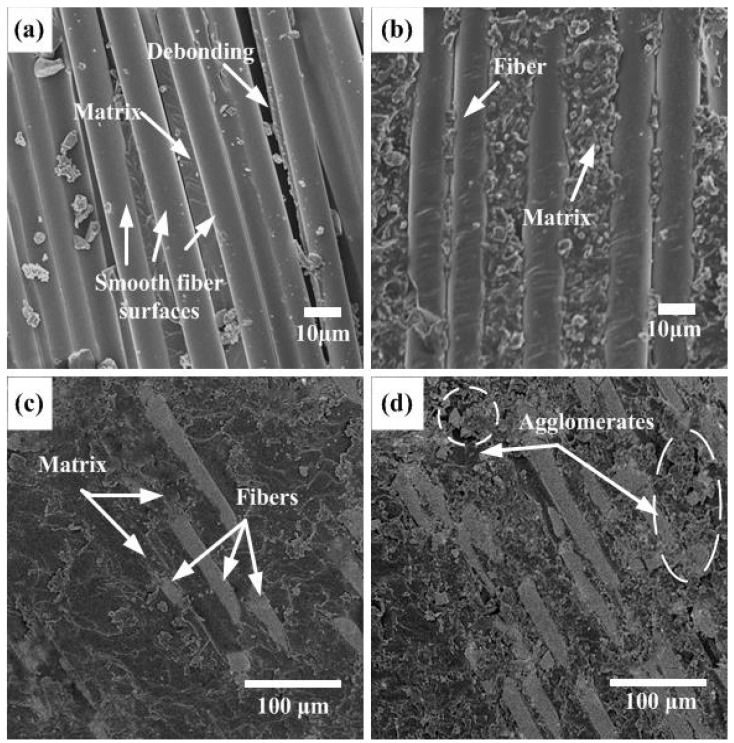
SEM images of the fiber/matrix surface on an HCS with: (**a**) 0 wt%: (**b**) 0.2 wt%: (**c**) 0.4 wt%: (**d**) 0.6 wt% NS content.

Figure 7 shows SEM micrographs of the adhesion between fibers/matrix with NS particle contents of (0, 0.2, 0.4, and 0.6 wt%). Basalt-fiber-reinforced HCS without NS has a smooth microscopic surface structure, as illustrated in Figure 7a. A smooth fiber surface without any matrix phase shows fiber/matrix debonding which is attributed to poor interfacial bonding. The fiber/matrix debonding reduces their load-carrying capacity and leads to a decrease in the stiffness of the HCS. Figure 7b shows the micro interface of the HCS modified by 0.2 wt% NS. There is no debonding between fiber/matrix, which attributes the matrix adhesion to be enhanced by adding NS particles. In the HCS with an NS content of 0.4 wt%, a good fiber/matrix interfacial bonding can be observed, and the surface of the fiber is covered with a layer of epoxy, as shown in Figure 7c. The uniform distribution of NS particles within the epoxy system results in an effective transfer of load between matrix and particles. Simultaneously, the freedom of fibers is limited by the epoxy matrix, which is coated on the fiber surface, leading to enhanced stiffness of the HCS. As shown in Figure 7d, in the case of 0.6 wt% NS, the particles tend to form agglomerates that reduce the NS/epoxy interfacial area. Thus, the stress transfer becomes less effective, and eventually, the stiffness of the NS-enhanced HCS decreases.

### 3.2. Performance Test of Tropocollagen-Inspired Basalt-Fiber-Reinforced HCS

#### 3.2.1. Compression Properties Analysis

**Figure 8:**
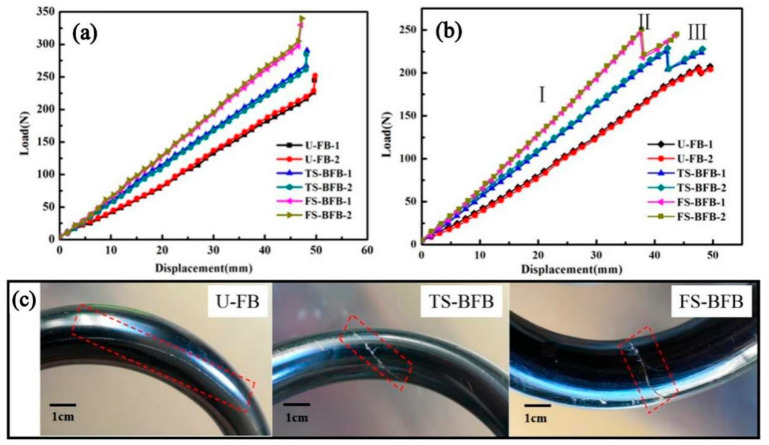
(**a**) Load-displacement curves before the HCS samples failure; (**b**) the failure curves of an HCS; and (**c**) the cracks in helical springs with different structures.

Figure 8a shows the load-displacement curves of three different HCS. It can be seen from the figures that the spring compression properties are basically stable. The mean spring stiffness, maximum compression load, and maximum compression displacement of the spring are listed in Table 2. As a result, the mean maximum compression load of HCS samples with U-FB, TS-BFB, and FS-BFB were 233 N, 264.5 N, and 301 N, respectively. The mean stiffness of the test HCS samples with U-FB, TS-BFB, and FS-BFB were 4.57 N/mm, 5.51 N/mm, and 6.55 N/mm, respectively. The maximum load of FS-BFB HCS increased by 29.2%, while the spring stiffness showed a 44% improvement compared with the U-FB HCS. The improvement of spring compression performance by the braided outer layer was also found to be only about 18% in other studies [39], which in some way reinforces the superiority of FS-BFB. This advantage may stem from the fact that the interwoven fiber bundles increase the transverse fiber content of the spring wire, thus improving the shear resistance of the wire. The more braided strands there are, the greater the transverse fiber content of the spring wire, the stronger the shear resistance of the spring wire. In addition, there were many interweaving points inside the interwoven fiber bundles. The existence of the interwoven points played a role in restraining the deformation of the fiber along the load direction. The less deformation of the wire spring under the same load, the higher the spring stiffness will be. More fiber strands lead to more interwoven points, which in turn lead to greater spring stiffness and stronger load-bearing capacity. The fluctuation of the curve of the braided fiber bundle structure is larger than that of the unidirectional fiber bundle, which may be caused by the uneven distance of the inner interwoven points of the spring wire.

#### 3.2.2. Failure Mode

Figure 8b shows the failure curves for each set of HCS after cyclic loading. The failure load of an HCS with U-FB, TS-BFB, and FS-BFB was 206 N, 225 N, and 246.5 N, respectively, which reduced by 11.6% (233 N), 14.9% (264.5 N), and 18.1% (301N) compared with that of maximum load before the HCS failure. It could be attributed to the release of residual stress inside the HCS. The maximum compression displacement of the HCS with FS-BFB and TS-BFB was 37.5 mm and 41.0 mm, respectively, which reduced by 21% and 13.7% that of U-FB (47.5 mm). The HCS with the structure of a braided-fiber bundle easily causes stress concentration at the interwoven points, resulting in a reduction in failure displacement.

As shown in Figure 8b, the compression failure process of an HCS can be divided into three stages. In stage I, the applied load was uniformly distributed over the spring wires, and the applied load increased almost linearly with the increase of compression. In stage II, the load decreased abruptly, and the phase was accompanied by a crisp sound. Once reaching the failure load, the fibers at interweave points cracked instantaneously, increasing the freedom of the fiber bundles. Both the decrease in load and the failure in the product at this stage were especially obvious for springs with braided-fiber bundle structures. In stage III, the wires of the HCS had been cracked. However, the load still increases with the increase of compression. This stage is different from the direct brittle fracture of metal springs. When one section of the spring wire was broken, the failure of this portion could be restrained via contacting the portion with its adjacent section of the spring wires, and the load was then transferred to and carried by other undamaged wires. As a result, the test curves kept on rising, implying the HCS still had carrying capacity and improved safety.

#### 3.2.3. Fracture Mechanism

Figure 8c illustrates the cracks of each structure of an HCS. The HCS with the U-FB structure began to crack at the matrix between the filament from the defect of resin, and this crack extended to the entire sample. Based on experimental observations, this phenomenon was not obvious in the spring structure of the braided-fiber bundle which is because the crack between the braided-fiber bundle was easier to be constrained by the interwoven points existing in both TS-BFB and FS-BFB structures. Because of the stress concentration, the cracks of TS-BFB and FS-BFB were more obvious than those of U-FB. Compared with the structure of TS-BFB, the HCS with the FS-BFB has a denser interwoven point. As a result, the crack size of the HCS with the FS-BFB was smaller than that of the TS-BFB HCS. In the process of spring compression, the internal stress on the spring wire would be released at the crack to reduce the generation of new cracks. As we know, the curvature inside the helical spring is larger than the outside, so the shear stress of the inner side of the spring is larger than that of the outer side during the compression. As a result, the cracks in the HCS structure began from the inner rim of its spring wire.

**Figure 9:**
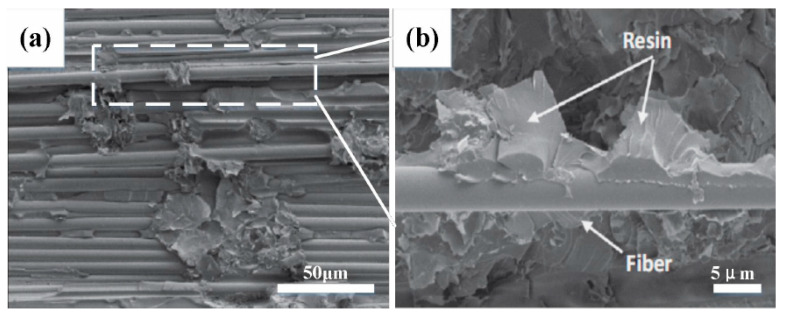
(**a**) Micro crack of braided fiber bundle HCS; (**b**) enlarged view of the white area in (**a**).

Figure 9a shows the SEM image at the crack. Figure 9b shows the enlarged view of the fracture area, showing the resin-enriched area is more brittle and prone to cracking, and the appearance of the crack leads to the debonding of the fiber, which reduces the compression performance of the composite spiral spring.

## 4. Conclusions

In this work, a new HCS was fabricated following the structure of collagen fibers, and firstly, the effects of different contents of NS on the properties of an HCS were investigated. The experimental results showed that the stiffness and fatigue resistance of an HCS were improved when an NS content of 0.4 wt% was added compared with the unmodified HCS. This is because the uniform dispersion of NS particles in the matrix enhances the fiber/matrix interfacial adhesion and transfers the load from the matrix to the NS particles effectively. When the content of NS exceeds 0.4 wt%, the particles tend to form agglomerates, leading to a decrease in the stiffness and fatigue properties of HCS. Basalt fiber/epoxy HCS with different fiber strands were prepared by bionic collagen fibers. The study showed that the spring stiffness and fracture load of an HCS with FS-FB were 6.55/mm and 246.5 N, respectively, which were 44% and 19.6% higher than those with U-FB, indicating that the bionic collagen fiber structure has great potential. The bionic HCS studied in this paper still needs a lot of work before it can replace metal springs, but it is important to explore suspension springs, and the next research study can further improve the performance of the springs by finding new particles or by borrowing other biological structures from nature.

## Figures and Tables

**Table 1:** Compression properties of helical springs.

NS content (wt%)	0	0.2	0.4	0.6
Compression (mm)	61.62	62.28	61.09	61.22
Stiffness (N/mm)	4.8	5.6	7.3	6.5

**Table 2:** Comparison of compression properties of an HCS with different spring wire structures.

Spring Wire Structures	Maximum Compression Load (N)	Maximum Compression Displacement (mm)	Stiffness (N/mm)
U-FB	233	47.5	4.57
TS-BFB	264.5	41.0	5.51
FS-BFB	301	37.5	6.55

## Data Availability

Not applicable.
